# A Novel Machine Learning-Based Strain Capacity Prediction Model of High-Grade Pipeline Girth Welds Using LightGBM

**DOI:** 10.3390/ma19040726

**Published:** 2026-02-13

**Authors:** Xiaoben Liu, Yanbing Wang, Yue Yang, Jian Chen, Pengchao Chen, Jiaqing Zhang, Dong Zhang

**Affiliations:** 1 National Engineering Research Center for Pipeline Safety, MOE Key Laboratory of Petroleum Engineering, Beijing Key Laboratory of Urban Oil and Gas Distribution Technology, China University of Petroleum-Beijing, Beijing 102249, China; 2 PipeChina Institute of Science and Technology, Tianjin 300450, China

**Keywords:** crack driving force, strain capacity, LightGBM, prediction model

## Abstract

Currently, the non-uniformity of girth weld positions makes their limit state a crucial determinant of pipeline safety. The design method based on the limit state is pivotal in ensuring the integrity and reliability of the pipeline system. Challenges often emerge when determining the limit states of girth welds using semi-empirical formula methods, primarily due to difficulties in accurately identifying influential factors. The quantitative impact of each influence parameter on the crack driving force and the results determined by the semi-empirical formula remain unclear. This study utilizes numerical simulation methods to systematically analyze the quantitative sensitivity laws of critical factors such as crack depth on the crack driving force to address this challenge. The findings revealed that the strength matching coefficient, crack depth, and misalignment are the most significant factors influencing the crack driving force, followed by crack length, softening rate, yield-to-strength ratio, internal pressure, and wall thickness. The effects of tensile strength and outer diameter are relatively minor. A comprehensive database of crack driving forces is constructed using a parameter matrix approach. Combined with the LightGBM machine learning algorithm, a full-scale prediction model for the strain capacity of pipeline girth welds is developed. Predictions for 18 sets of wide-plate test results from the literature confirm the high accuracy of the prediction model, with a prediction accuracy of 6.48%. This research provides a robust reference for accurately determining the limit state of pipeline girth welds and effectively meets the demands of rapidly advancing welding technologies and increasingly complex service environments.

## 1. Introduction

With the continuous expansion of the global pipeline network, the pipeline system is progressively evolving towards larger diameters and high pressures. Ensuring the safe operation of pipelines with high steel grades, large diameters, and significant wall thicknesses presents a formidable challenge. The girth weld area represents the most vulnerable link in the entire pipeline system. Cracks that originate and propagate in the girth weld region are the predominant failure modes for these welds. Numerous pipeline accidents have resulted from such failures in recent years, leading to severe consequences [1,2,3,4].

Accurately determining the ultimate strain capacity is essential to ensure the safety of pipeline girth welds. The most effective method to achieve this is through full-scale pipeline testing. However, the considerable expense of such tests has become a significant obstacle to research in this field. Researchers combined limited experimental studies (full-scale tensile tests and wide plate tensile tests) with comprehensive finite element simulations and prediction methods to develop a predictive model for the ultimate state of girth welds, which has become the prevailing approach [5]. Various international research institutions successfully introduced prediction models for the ultimate state, primarily in semi-empirical formulas and neural networks. Ghent University [6,7,8] introduced in 2004 a semi-empirical strain capacity prediction model based on the statistical analysis of 480 wide plate tensile test datasets. However, the model has limitations, as it does not account for internal pressure, misalignment, and fracture toughness. The Pipeline Research Council International (PRCI) [9,10,11,12,13] developed semi-empirical formulas in 2006 using finite element methods considering surface crack and embedded crack conditions in pipeline girth welds. However, the model is unsuitable for low-strength mismatch welds and fails to consider the softening effect in the heat-affected zone, thus maintaining certain limitations. In 2011, ExxonMobil [14] unveiled the first-generation ExxonMobil ultimate strain capacity prediction model using static crack finite element simulations based on crack instability growth failure criteria. This model was enhanced in 2014 [15] by incorporating the Gurson–Tvergaard–Needleman (GTN) damage model to mitigate the shortcomings of the initial version. Also in 2011, CRES [16,17,18,19,20] proposed the CRES ultimate tensile strain calculation model based on PRCI’s initial model, concentrating on Gas Metal Arc Welding (GMAW) and Flux Cored Arc Welding (FCAW) processes. Despite extensive numerical simulations based on static cracks, the model did not address low-match conditions. In 2022 [21], the research team at China University of Petroleum developed a restraint-corrected Type I fracture failure criterion for pipeline girth welds, establishing the prediction model for the ultimate strain capacity of cracked girth welds. However, the model presumes relatively large crack sizes, limiting its applicability.

Determining the ultimate state using prediction models holds great importance. For existing pipelines, it allows for predictions of girth weld load-bearing capacities based on various factors. For pipelines under construction, it offers essential safety guidance. PRCI [20] identified in 2011 that factors affecting the ultimate load-bearing capacity of girth welds include weld metal tensile properties, crack size, and structural dimensions. Then, they determined how these factors influence crack driving forces. Although methods for determining the ultimate state of girth welds based on predictive model inputs sometimes exhibit high accuracy, their application conditions are relatively stringent, and precise values are challenging to determine, especially concerning welding factors. Records for older pipelines are often lost, and quantifying factors for new pipelines pose similar challenges [22,23,24]. Prediction models that rely on semi-empirical formulas overlook considerations for high steel grades and low match situations, necessitating an expansion of their scope of applicability.

Accordingly, identifying the critical factors that significantly influence the initiation of cracks in pipeline girth welds is crucial for ensuring the accuracy of ultimate state assessments, particularly when faced with challenges from incomplete information. Therefore, conducting sensitivity analysis on crack driving forces becomes particularly necessary. This study analyzes the key factors affecting the driving forces of cracks in pipeline girth welds and further quantifies the sensitivity of each factor’s impact on crack driving forces. Using the Light Gradient Boosting Machine (LightGBM) algorithm and incorporating data from 10,935 sets of weld centerline crack driving forces, a prediction model for crack driving forces is established to assess and enhance the safety performance of pipeline structures. These analytical findings are significant for optimizing pipeline design and construction strategies, providing a scientific foundation for engineering decisions, and significantly enhancing pipeline safety and reliability. It should be noted that the subsequent prediction model established in this study is developed for girth welds with pre-existing crack defects. The model is dependent on the input of crack depth and crack length, and the FE simulation data used to build the database all target cracked welds, so the model has no predictive ability for crack initiation and is not suitable for the analysis of no-crack girth weld conditions.

## 2. Finite Element Simulation of a Crack Driving Force

### 2.1. Pipeline Geometric Structure Modeling

#### 2.1.1. Geometric Model

**Figure 1:**
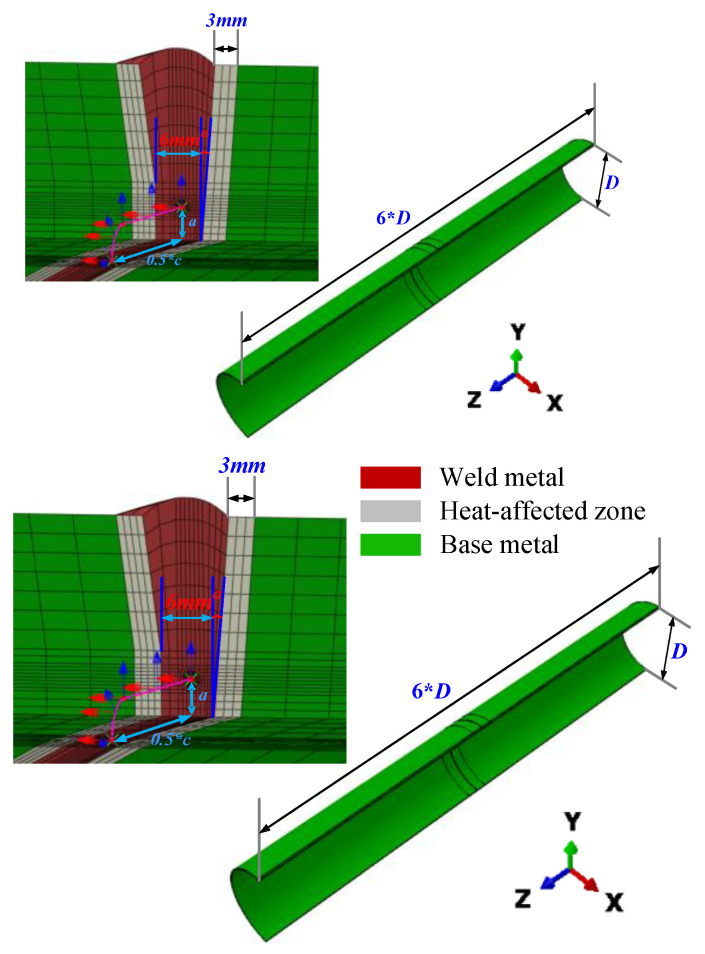
Full-size pipe geometry model diagram.

Typically, full-scale pipelines feature a cylindrical shell structure connected by welding, with the central part being the girth weld. Ignoring any out-of-roundness, both the pipeline and the circumferential crack exhibit symmetry. A half-scale pipeline model was established to simulate the response of the base metal weld assembly to axial loads more accurately, extract the driving force of circumferential cracks more simply and efficiently, reduce the computational workload of finite element models, and enhance computational efficiency. The symmetry plane is perpendicular to the circumferential crack face at the crack’s center point. The pipeline model has a diameter (*D*) of 1422 mm, an axial length of 6*D*, and a wall thickness (*t*) of 21.4 mm. The girth weld is located at the center of the pipeline model, using an automatic welding process. The positions of the base metal, heat-affected zone, and weld are clearly distinguished. The heat-affected zone spans 3 mm, the weld length is 6 mm, the weld bevel angle *α* is 5°, the crack depth is a, and the crack length is c (Figure 1).

#### 2.1.2. Mesh Subdivision

**Figure 2:**
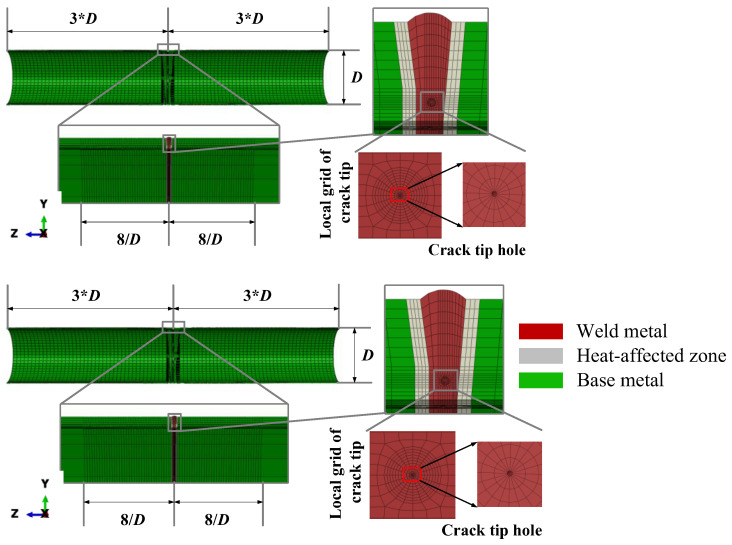
Full-scale pipeline mesh division and crack tip mesh refinement schematic diagram.

This study utilizes static crack simulation techniques to develop a numerical model, which proves highly effective in analyzing the variations in crack tip driving forces under conditions of elastoplastic large deformation. Compared to the GTN model, which is grounded in damage theory or void growth theory, the static crack model offers a significant advantage in computational efficiency. The effectiveness of mesh division is evident in its dual role of enhancing computational speed while maintaining high calculation accuracy. The research focuses on the mechanical response of the crack tip region within the weld area of full-scale pipelines with cracked defective girth welds. Therefore, local mesh refinement was performed in the left and right 8/*D* regions adjacent to the weld to detect potential high-stress concentrations during loading (Figure 2).

A spiderweb-like mesh division strategy was implemented near the crack surface, incorporating a keyhole model to describe the blunting behavior at the crack tip. This strategy optimized the extraction of crack driving force calculation results and ensured precision. The use of eight-node hexahedral elements and six-node wedge elements was strategic; eight-node hexahedral elements ensured sufficient calculation accuracy in the primary regions of the model, whereas six-node wedge elements were utilized in transitional areas and near the crack tip to accommodate boundary conditions and capture stress concentration phenomena. This mixed-element model ensures computational efficiency while accommodating the complexities of simulating crack tip stress fields.

The choice of mesh density significantly impacts the accuracy of the calculation results and the efficiency of computational resource utilization. This study employs a variable density mesh division strategy, incorporating a high-density mesh near the critical crack tip area with a minimum grid size of 0.04 mm to precisely capture the stress–strain response at the crack tip. The configuration comprises 20 grids in the circumferential direction and ten layers radially, designed to accurately resolve the stress–strain field at the crack tip while preserving overall model computational efficiency.

#### 2.1.3. Boundary Conditions and Loading

**Figure 3:**
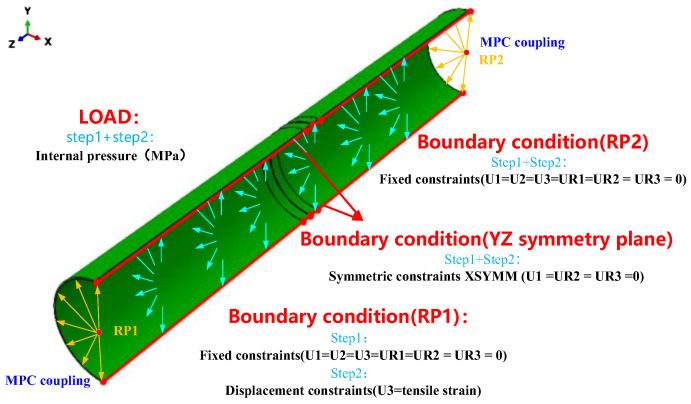
Full-size pipeline load and boundary condition diagram.

This study establishes accurate boundary conditions on the 1/2 pipeline model to simulate the service environment of the actual pipeline. It applies the load conditions typical of the actual pipeline to ensure that the model accurately reflects the mechanical response of the pipeline under actual operating conditions (Figure 3).

Since the full-scale pipeline model in this study is a 1/2 model, symmetric boundary conditions were applied along the YZ symmetry plane. This research examines the mechanical responses of cracks at the weld centerline as strain increases under internal pressure and tensile load in full-scale pipelines. Point-to-face coupling constraint (MPC) technology was employed to link the cross-sections at both ends of the pipeline model to corresponding reference points, accurately simulating the actual loading conditions of the pipeline. A uniform force condition across the entire pipeline cross-section can be effectively simulated by exerting loads on these reference points. Considering that pipelines subjected to displacement loads typically operate under steady pressure, two analysis steps were determined in this study: one to apply internal pressure within the pipeline to simulate the conditions it experiences during actual operations and another to set reference points at both ends of the pipeline, fixing reference point 2 while applying a displacement load to reference point 1. The displacement amount was calculated based on the total tensile strain and the model’s length. This study adopted 1/2 model symmetry boundaries and MPC technology to enhance the scientific validity of the loading conditions for the full-scale pipeline, thus ensuring the scientific accuracy of the model results.

#### 2.1.4. Grid Independence Verification

This study conducts grid independence verification to ensure the reliability of the finite element model. The parameters of the verification model include a pipe diameter of 1422 mm, wall thickness of 21.4 mm, strength matching coefficient of 1.0, yield ratio of 0.89, crack length of 50 mm, crack depth of 4 mm, softening rate of 0.0, internal pressure of 12 MPa, and misalignment of 0 mm.

**Figure 4:**
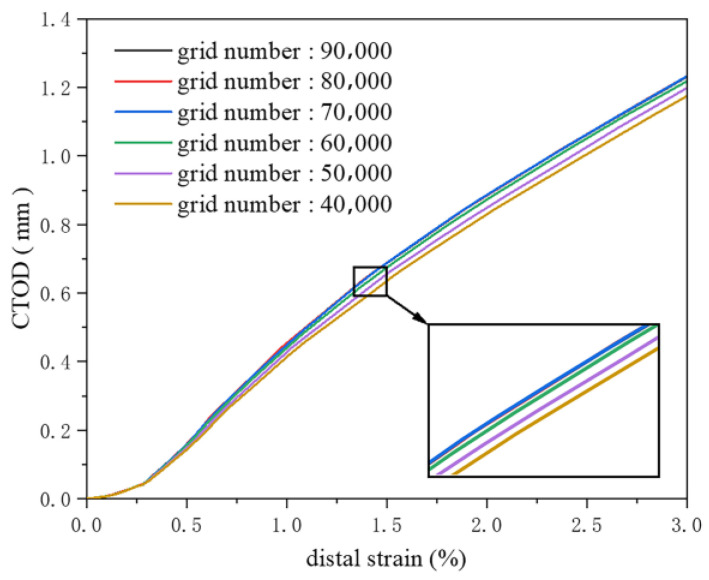
Grid independence verification.

As the number of elements ranges from 40,000 to 70,000, the crack driving force increases gradually with the rise in element count. Coarser meshes failed to precisely capture the stress concentration at the crack tip, leading to an underestimation of the crack driving force. With increasing numbers of elements, the finer finite element model captures the stress concentration at the crack tip more accurately. When the number of elements exceeds 70,000, the crack driving force curve stabilizes and remains essentially constant, indicating that at this level of refinement, the finite element model is sufficiently detailed to reflect the stress distribution at the crack tip. Further increases in the number of elements have a negligible impact on the results (Figure 4). Accordingly, once a certain level of refinement is achieved, the computational results no longer depend on changes in the number of elements, thus verifying the mesh independence of the model. This study selects a simulation with approximately 70,000 elements to ensure the accuracy of the computational results while enhancing computational efficiency.

### 2.2. Material Properties

Ensuring the accuracy and reliability of the finite element model requires a precise description of material properties.

#### 2.2.1. Base Metal

The smooth dome shape is recognized as the most accurate depiction of the stress–strain relationship, and the CSA Z662 [25] constitutive model is one of the most representative of this. The quantitative relationships between the CSA Z662 material’s stress–strain curve, the yield ratio, and the hardening characteristics of the base metal and yield ratio are clarified.

(1) $$\varepsilon =\sigma /E+\left(0.005-\sigma_{y}/E\right){\left(\sigma /\sigma_{y}\right)}^{n}$$

(2) n=3.14/(1−λ)

where *ε* is the strain, *σ* is the stress (MPa), *E* is the elastic modulus (MPa), *σ_y_* is the yield strength of the material (MPa), *n* is the hardening coefficient of the base metal, and *λ* is the yield ratio of the pipe.

#### 2.2.2. Heat-Affected Zone

The heat-affected zone is also characterized using the CSA Z662 stress–strain constitutive model. The tensile strength differs from that of the base metal. The existence of a softening rate *μ* is acknowledged. The tensile strength of the base metal, when multiplied by (1 − *μ*), determines the tensile strength of the heat-affected zone. Other factors remain identical to those of the base metal. The softening rate of the heat-affected zone is defined as follows:

(3) $$\mu =1-\sigma_{tHAZ}/\sigma_{tb}$$

where $\sigma_{tHAZ}$ is the tensile strength of the heat-affected zone (MPa), and $\sigma_{tb}$ is the tensile strength of the base metal (MPa).

#### 2.2.3. Weld

The mechanical properties of welds differ from those of the base metal and heat-affected zone (HAZ), with various welding processes typically resulting in unique mechanical characteristics for the welds. Wu Kai et al. [26] conducted a study on the girth welds of the China-Russia East-Route X80 pipeline, employing complete weld metal tensile tests using notched round bars to determine the mechanical properties of the welds. The results indicate that the weld exhibits a yield platform before 2% strain. The stress–strain relationship before 2% strain is also described by the CSA Z662 stress–strain constitutive model. Based on experimental data, Wu Kai et al. established a linear relationship be-tween the weld’s yield strength and tensile strength, the weld metal yield-to-strength ratio, and the hardening coefficient obtained from the fitting.

(4) $$\sigma_{yw}=1.025\times \sigma_{tw}-58.7\mathrm{MPa}$$

(5) $$n_{w}=2.669/{\left(1-Y/T\right)}^{0.9765}$$

where $\sigma_{yw}$ is the yield strength of the weld (MPa), $\sigma_{tw}$ is the tensile strength of the weld (MPa), $n_{W}$ is the hardening coefficient of the weld, and *Y*/*T* is the yield-to-strength ratio of the weld.

The engineering stress–strain constitutive model for the weld metal is presented as follows:

(6) $$\left\{\begin{array}{l} \varepsilon =\frac{\sigma }{E}\;\;\;\;\;\;\;\;\;\;\;\;\;\;\;\;\;\;\;\;\;\;\;\;\;\;\;\;\;\;\;\;\;\;\;\;\;\;\;\;\;\;\;\;\;\;\;\;\;\;\;\;\;\;\;\;\;\;\;\;\;\;\;\;\;\;\;\;\;\;\varepsilon <\sigma_{y}/E\\ \sigma =\sigma_{yw}\;\;\;\;\;\;\;\;\;\;\;\;\;\;\;\;\;\;\;\;\;\;\;\;\;\;\;\;\;\;\;\;\;\;\;\;\;\;\;\;\;\;\;\;\;\;\;\;\;\;\;\;\;\;\;\;\sigma_{yw}/E<\varepsilon <2\%\\ \varepsilon =\sigma /E+\left(0.005-\sigma_{yw}/E\right){\left(\sigma /\sigma_{yw}\right)}^{n_{w}}+0.015\;\;\;\;\;\;2\%<\varepsilon \end{array}\right.$$

### 2.3. Finite Element Model Accuracy Validation

Full-scale pipeline tests subject the entire pipe section with girth welds to combined bending and internal pressure, capturing the synergistic effects of material heterogeneity, flaw geometry, and structural deformation, thus most accurately representing the in-service conditions of pipelines. To validate the finite element model, full-scale tests are required.

In 2024, a full-scale four-point bending test on girth welds of D1219 × 22 mm X80 pipelines was conducted. Details of the test material, test procedure, measurement system, and test results have been described in our previous publications [27]. In this study, only the key test information is presented, with the focus on comparing the test results with those calculated by the finite element model established in this research.

**Figure 5:**
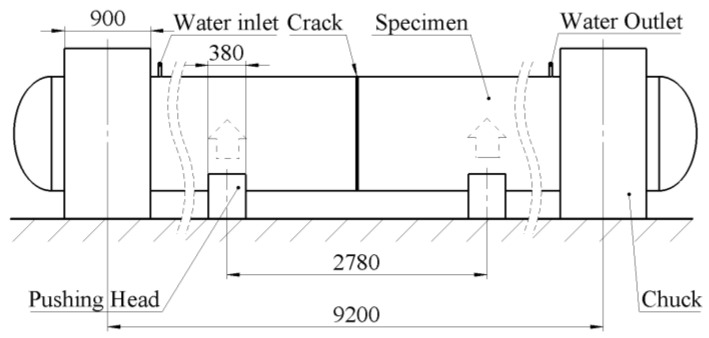
Size and loading diagram of the test device.

**Figure 6:**
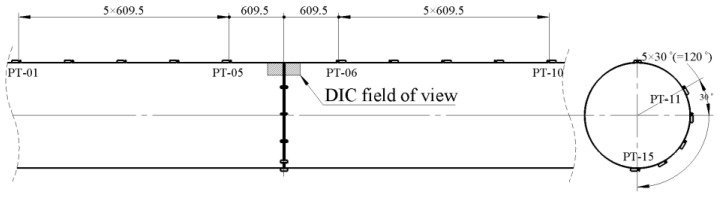
Schematic diagram of strain gauge arrangement.

The total length of the test pipeline was 1040 mm, which was cut into two sections from the middle and then welded. End caps were welded to both ends of the pipeline to form a closed pressure vessel. A circumferential crack was machined on the top of the pipeline, with a crack depth of 15 mm and a circumferential length of 300 mm. The test involved phased application of water pressure and bending load. Firstly, pressures of 5, 8, and 10 MPa were applied to the specimen in stages, with short-term pressure holding at each stage. Then, the ram applied an upward displacement at a speed of 1 mm/min until the specimen fractured and failed, ejecting high-pressure water. Subsequently, pressure unloading was performed, and the ram was displaced downward to the origin (Figure 5). During the test, the load–displacement curve of the testing machine was recorded synchronously, and strain gauges and digital image correlation (DIC) were used for strain measurement (Figure 6).

**Figure 7:**
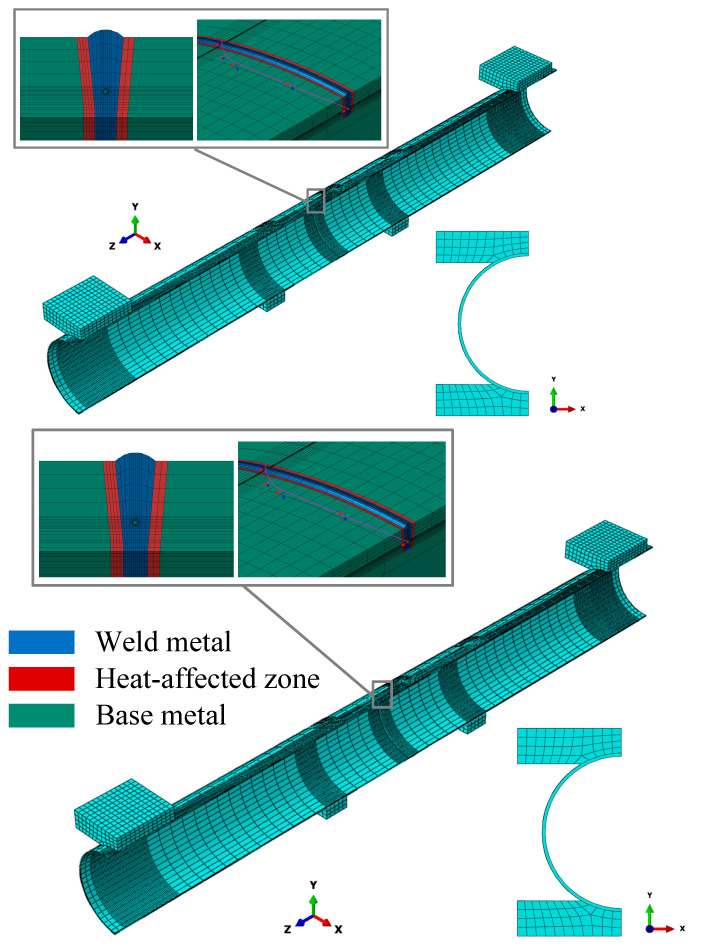
Finite element model of the four-point bending test.

Using the crack modeling method described in the previous section, a half-pipe geometric model was established in a 1:1 scale with the actual test dimensions. To accurately simulate the bending load applied to the pipeline, rigid body structural models of the ram and chuck were constructed. Surface-to-surface contact was set between these rigid models and the outer surface of the pipeline: hard contact was adopted in the normal direction, and the tangential friction coefficient was set to 0.3 (Figure 7). The model loading was performed in three steps: Step 1: Apply an internal pressure of 10 MPa to the pipeline. Step 2: The ram applies a displacement consistent with that in the experiment. Step 3: Unload the water pressure and bending load.

**Figure 8:**
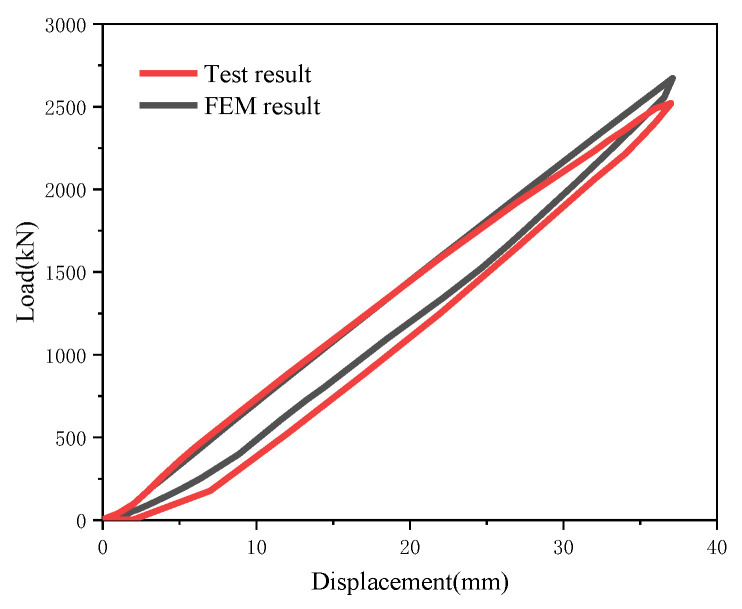
Comparison of load displacement curves between FEM and test.

The comparison between the simulated ram load–displacement curve and the experimental counterpart reveals that the two curves exhibit a high degree of consistency (Figure 8). The maximum ram load obtained from the experiment is 2522.75 kN, while the maximum ram load derived from the simulation is 2610.32 kN, with a relative error of 3.47%.

**Figure 9:**
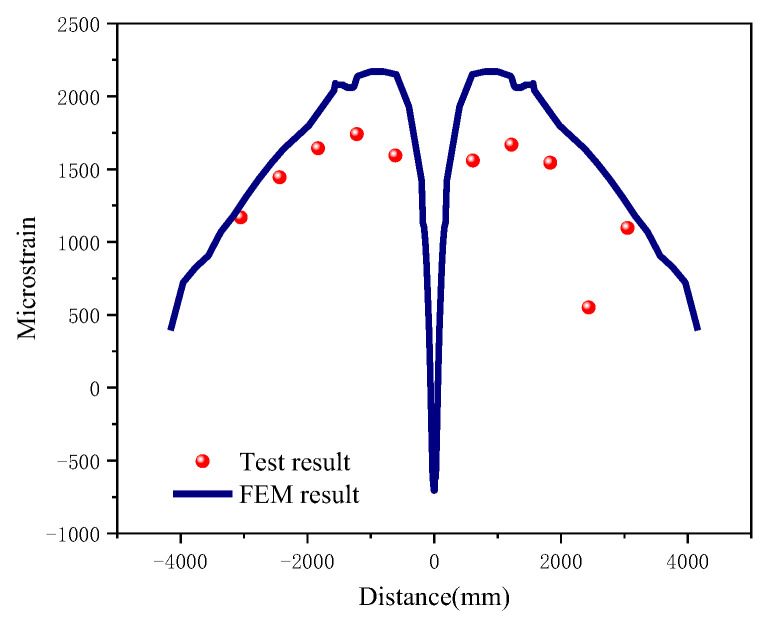
Comparison of FEM strain and strain gauge results.

**Figure 10:**
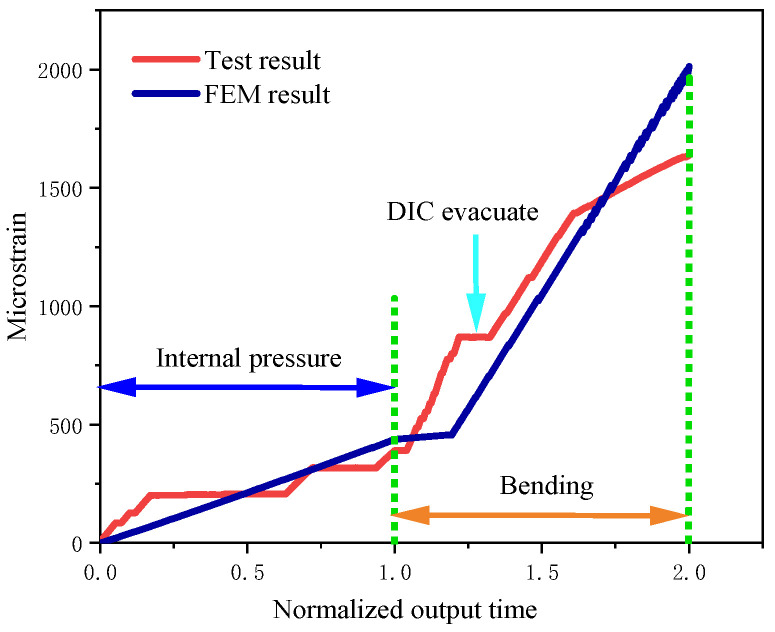
Comparison of axial strain at the position 1D away from the weld between FEM and test.

Axial strain data along the top path of the pipe at the moment of weld failure was extracted and compared with the strain gauge measurement results. The overall trend of the simulated critical strain curve was consistent with the strain values recorded in the experiment, with an average error of 14.05% (Figure 9). Furthermore, the strain–time curves of elements located at a position 1*D* away from the crack surface were extracted and compared with the curves recorded by the experimental strain gauges. During the internal pressure loading stage, stepwise pressure application was adopted in the experiment, resulting in a step-like variation in the curves. During the bending loading stage, at the initial moment of loading, the ram had not yet contacted the pipe surface; once the ram came into contact with the pipe, the strain showed linear growth, and the experimental and simulated results exhibited good consistency (Figure 10).

**Figure 11:**
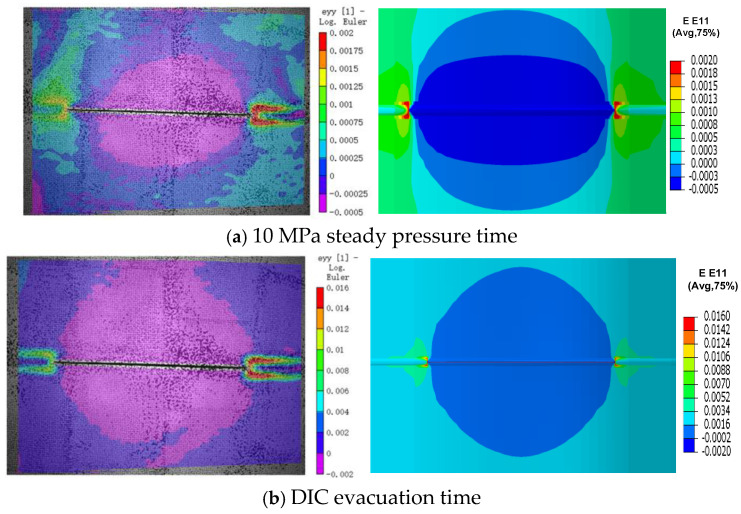
Comparison of strain cloud map between FEM and test.

Considering that the water column generated by the rupture of a pressurized pipeline might cause damage to the entire DIC equipment, the DIC equipment was evacuated from the test site before the girth weld ruptured. Therefore, the strain distribution at the moment when internal pressure loading was completed and at the moment when the DIC equipment was evacuated was compared with that from the finite element model. It can be seen that the comparison results of the overall contour maps are favorable, and the local strain results show little difference (Figure 11).

Through the modeling method adopted in this study, a comprehensive comparison was conducted between the results derived from this method and the full-scale experimental results. This comparison directly verifies the accuracy of the fixed crack method adopted in this study for simulating crack defects and the “key hole” model for capturing the stress concentration at the crack tip.

## 3. Analysis of Influencing Factors of Crack Driving Force

### 3.1. Pipe Outer Diameter

**Figure 12:**
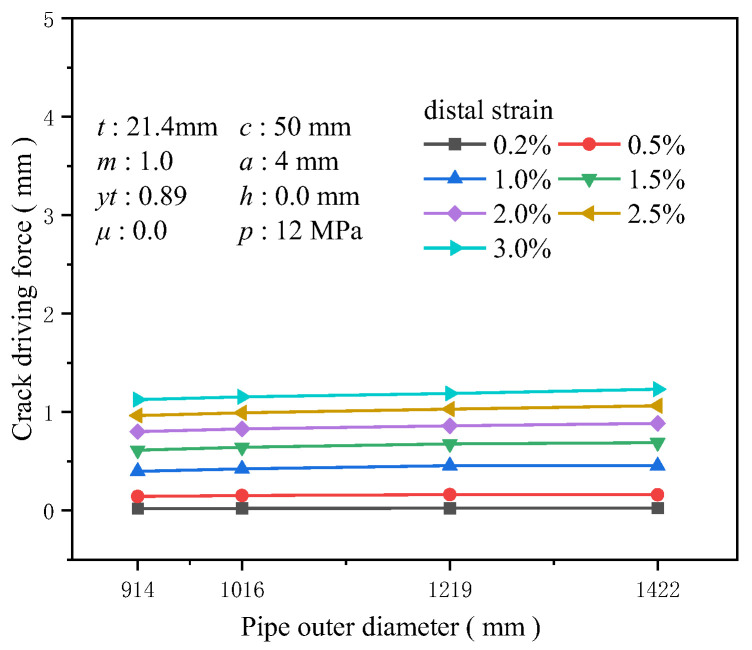
The crack driving force when the crack reaches various tensile strains under different outer diameter conditions.

**Figure 13:**
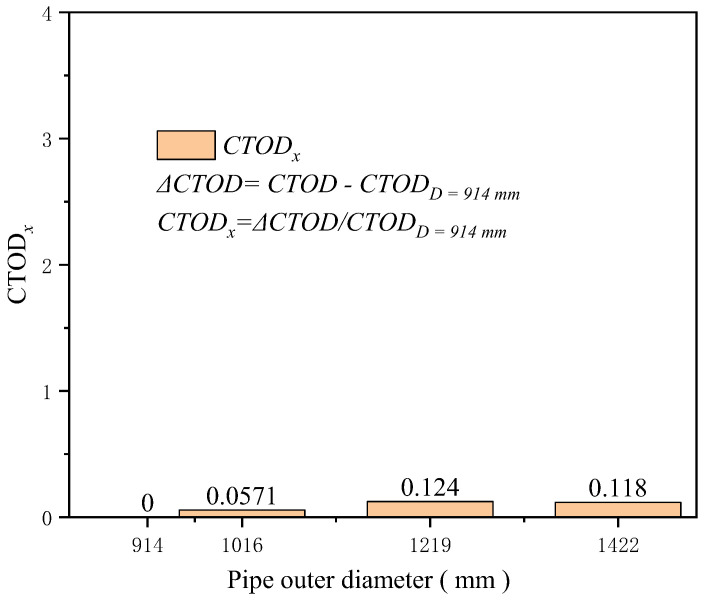
Under the condition of 0.5% distal strain, the change in pipe diameter causes an increase in driving force.

The wall thickness was set to 21.4 mm, and common large-diameter dimensions for high-grade steel pipelines were chosen for the outer diameters 914, 1016, 1219, and 1422 mm to investigate the influence of the pipeline’s outer diameter on the crack driving force. Although maintaining other factors constant, it was found that the crack driving force positively correlates with the pipeline’s outer diameter. A larger outer diameter implies that under the same strain conditions, the circumferential surface area of the pipeline subjected to stress increases, and the axial stress generated by internal pressure and additional loads also rises, intensifying the stress concentration in the weld area. However, the numerical results indicate that diameter is one of the least sensitive factors affecting the crack driving force. At 3% tensile strain, the increase in the crack driving force for a diameter of 1422 mm compared to 914 mm is only about 0.11 mm (Figure 12). At 0.5% tensile strain, the increase for a diameter of 1422 mm compared to 914 mm is just 0.118 times that at 914 mm (Figure 13).

### 3.2. Wall Thickness

**Figure 14:**
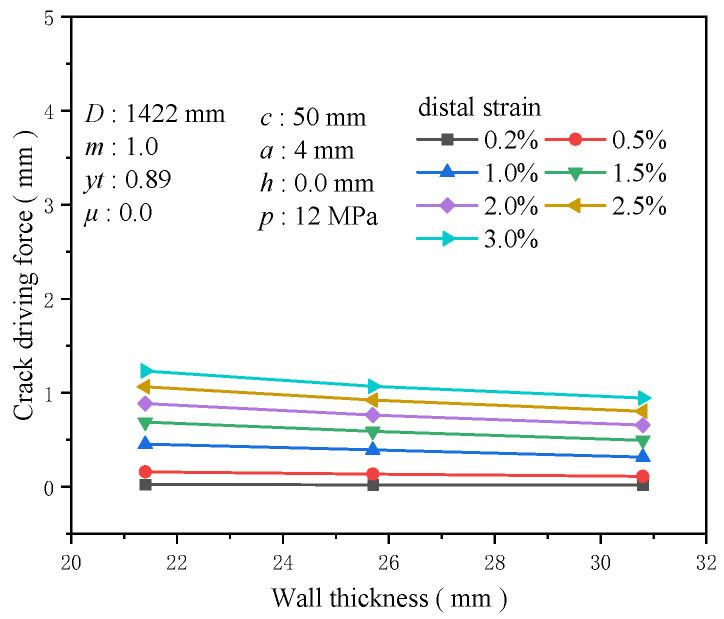
The crack driving force when the crack reaches various tensile strains under different wall thickness conditions.

**Figure 15:**
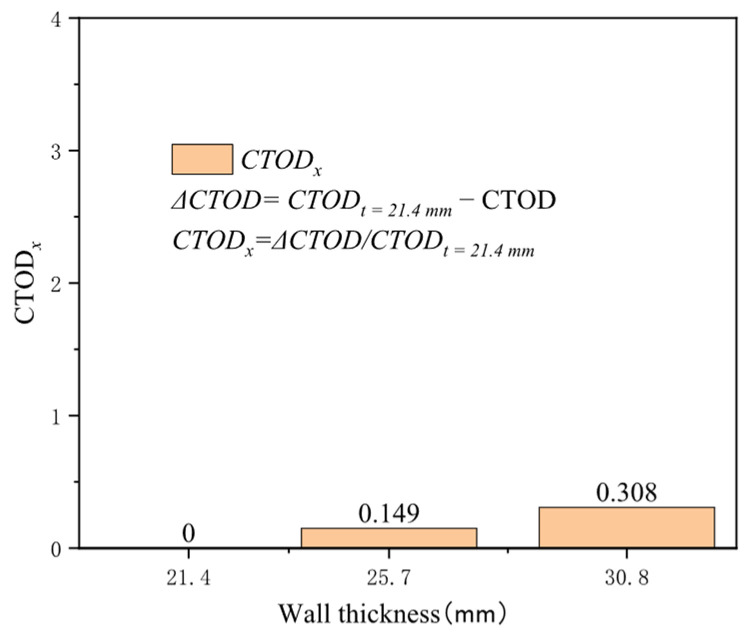
Under the condition of 0.5% distal strain, the change in wall thickness causes an increase in driving force.

Pipeline wall thickness is an essential structural parameter directly affecting axial stress distribution on the pipeline wall thickness. It is a critical structural parameter directly influencing axial stress distribution on the pipeline wall surface. With a constant outer diameter of 1422 mm, three common wall thicknesses for high-grade steel were selected: 21.4, 25.7, and 30.8 mm. Increasing the wall thickness typically enhances the pressure vessel’s load-bearing capacity and alleviates local stress concentrations. In the early stages of minor strain, the crack driving force’s sensitivity to wall thickness is limited but increases under extensive strain conditions. The increase in crack driving force when the wall thickness is raised from 21.4 mm to 30.8 mm at 0.2% tensile strain is approximately 0.006 mm, and at 3% tensile strain, it is about 0.288 mm (Figure 14). The effect of diameter on the crack driving force is nearly linear under the same tensile strain conditions. At 0.5% tensile strain, the increase in the crack driving force for a wall thickness of 25.7 mm compared to 21.4 mm is 0.149 times that at 21.4 mm, and for 30.8 mm, the increase reaches 0.308 times (Figure 15). Wall thickness is a more sensitive structural parameter affecting crack driving force than diameter.

### 3.3. Strength Matching Coefficient

**Figure 16:**
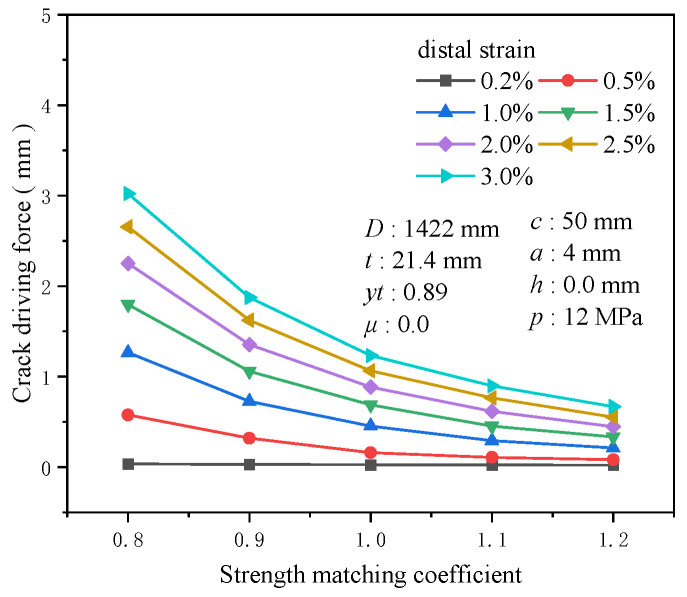
The crack driving force when the crack reaches various tensile strains under different strength matching coefficient conditions.

**Figure 17:**
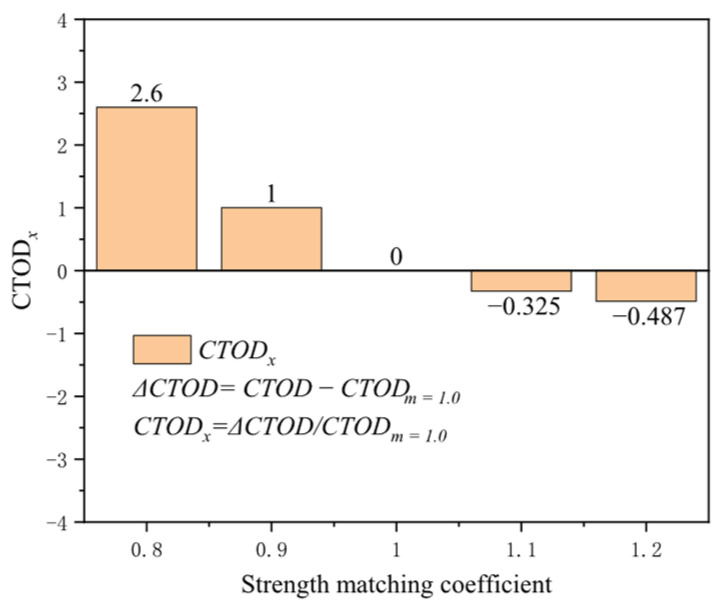
Under the condition of 0.5% distal strain, the change in strength matching coefficient causes an increase in driving force.

The strength matching coefficient typically denotes the ratio of the weld’s tensile strength to that of the base metal, reflecting the strength level in the weld area and impacting the crack driving force at the girth weld location. The strength matching coefficient’s influence on the crack driving force is negligible at a tensile strain of 0.2%, with a mere 0.014 mm difference between high strength (*m* = 1.2) and low strength (*m* = 0.8). As strain increases, the sensitivity of the strength matching coefficient to the crack driving force becomes more pronounced. At 3% tensile strain, the difference reaches 2.35 mm between high strength (*m* = 1.2) and low strength (*m* = 0.8) (Figure 16). The crack driving force exhibits high sensitivity to the strength matching coefficient at low matching levels. At 0.5% tensile strain, the increase in the crack driving force for low strength (*m* = 0.8) compared to equal strength (*m* = 1.0) is 2.6 times that of the crack driving force at equal strength (*m* = 1.0), while for high strength (*m* = 1.2) compared to equal strength (*m* = 1.0), the increase is only 0.48 times (Figure 17). Compared to other material property factors, the strength matching coefficient emerges as one of the most influential.

### 3.4. Tensile Strength

**Figure 18:**
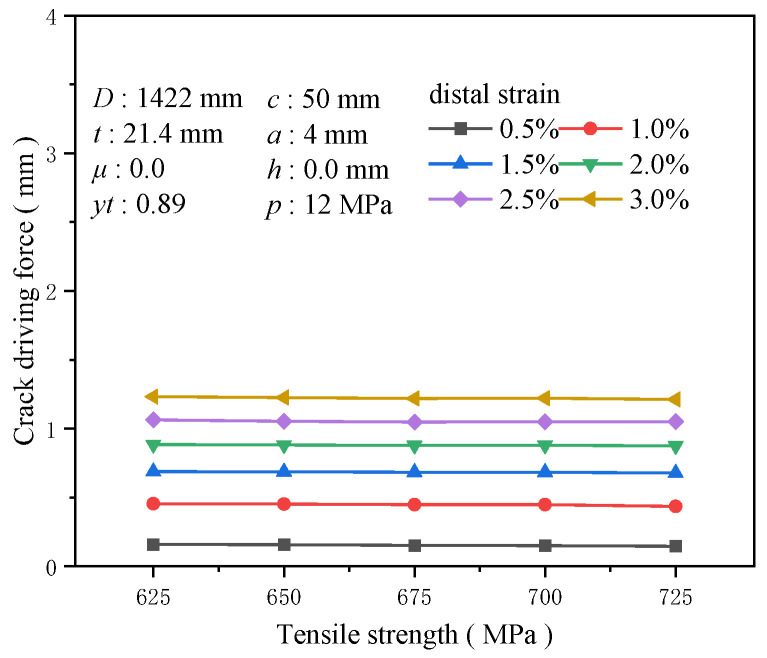
The crack driving force when the crack reaches various tensile strains under different tensile strength conditions.

**Figure 19:**
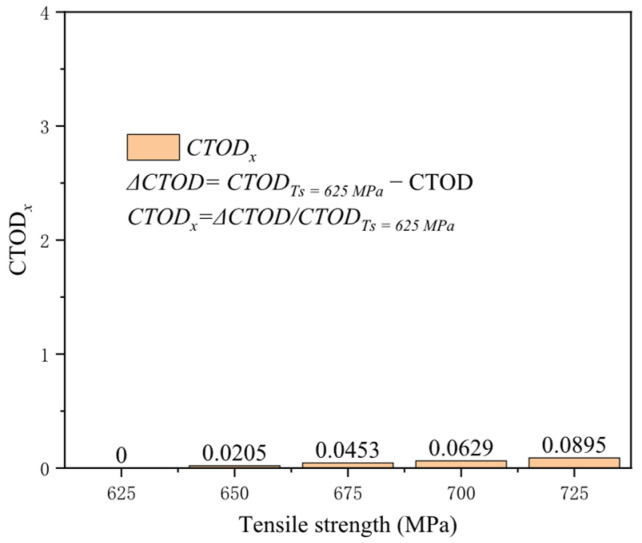
Under the condition of 0.5% distal strain, the change in tensile strength causes an increase in driving force.

Tensile strength, the critical value at which uniform plastic deformation transitions to local concentrated plastic deformation, represents the material’s capacity to withstand maximum tensile stress. Typically, materials with higher tensile strengths exhibit superior plastic deformation capabilities, enhancing their resistance to crack propagation under external loads. With a yield-to-tensile ratio of 0.89, the impact of varying tensile strengths on the crack driving force was assessed (Figure 18). Although an increase in tensile strength somewhat reduces the crack driving force, the effect remains extremely limited. At a tensile strain of 3%, elevating the tensile strength from 625 to 725 MPa diminishes the crack driving force by only 0.019 mm. At a tensile strain of 0.5%, the reduction in the crack driving force for tensile strength of 725 MPa compared to 625 MPa is only 0.08 times that at 625 MPa (Figure 19). As advancements in steel pipe materials continue, high yield strength and tensile strength have become industry standards, but only increasing tensile strength does not ensure enhanced crack resistance in welds. Thus, attention must also be directed toward the yield-to-tensile ratio and other relevant mechanical performance indicators.

### 3.5. Yield-to-Tensile Ratio

**Figure 20:**
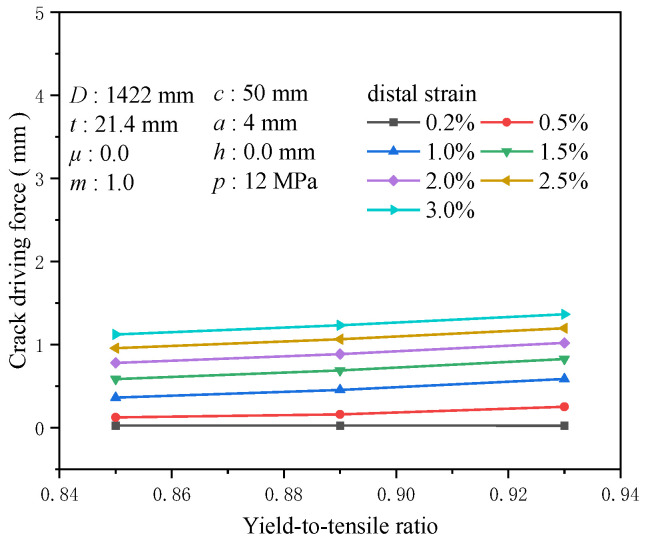
The crack driving force when the crack reaches various tensile strains under different yield-to-tensile ratio conditions.

**Figure 21:**
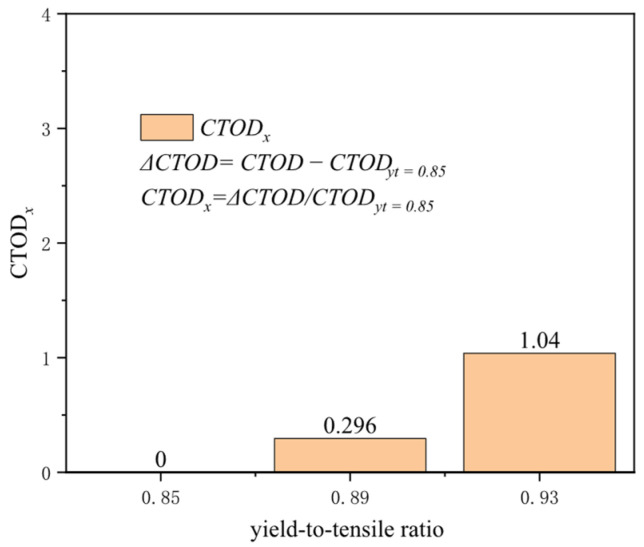
Under the condition of 0.5% distal strain, the change in yield-to-tensile ratio causes an increase in driving force.

The yield-to-tensile ratio, representing a material’s yield strength to its tensile strength, indicates its capacity to undergo plastic deformation and sustain additional strain after reaching the yield point. Materials with a high yield-to-tensile ratio exhibit a relatively limited ability to endure further strain post-yield. Materials that demonstrate inferior plastic deformation capabilities for girth welds often result in stress concentrations at the crack tip, which cannot be effectively dissipated, facilitating crack propagation. With a tensile strength of 625 MPa and common yield-to-tensile ratios of 0.85, 0.89, and 0.93, variations in the yield-to-tensile ratio exhibit negligible influence on the crack driving force at a tensile strain of 0.2%. The influence of the yield-to-tensile ratio on the crack driving force becomes apparent around the yield point. At a tensile strain of 0.5%, altering the yield-to-tensile ratio from 0.85 to 0.93 results in a change in the crack driving force by approximately 0.128 mm, and at a tensile strain of 3%, this alteration reaches about 0.242 mm (Figure 20). When the tensile strain escalates to 0.5%, the increase in crack driving force for a yield-to-tensile ratio of 0.93 compared to 0.85 is 1.04 times the crack driving force at a yield-to-tensile ratio of 0.85. In contrast, the enhancement for a yield-to-tensile ratio of 0.89 compared to 0.85 is merely 0.296 times (Figure 21). Hence, particular attention must be given to the impact of the yield-to-tensile ratio when it exceeds 0.89. The yield-to-tensile ratio is a critical parameter affecting the overall performance of welds and materials. Proper management of the yield-to-tensile ratio is essential for optimizing structural design and enhancing the safety and reliability of girth welds.

### 3.6. Crack Length

**Figure 22:**
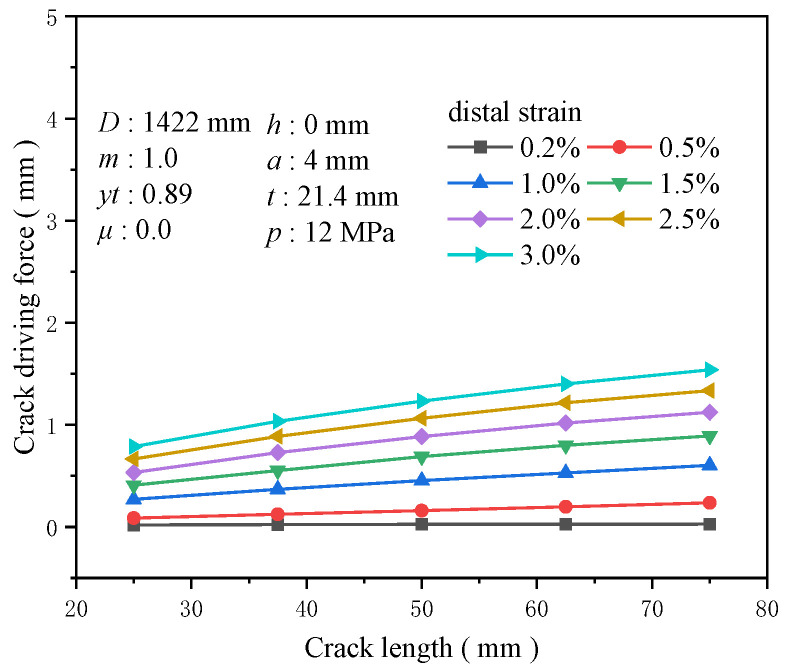
The crack driving force when the crack reaches various tensile strains under different crack length conditions.

**Figure 23:**
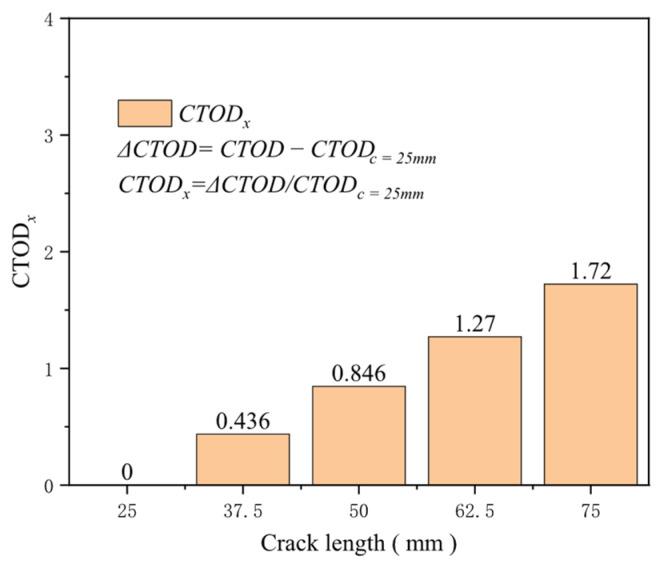
Under the condition of 0.5% distal strain, the change in crack length causes an increase in driving force.

The size of initial defects, particularly the initial crack length, significantly affects structural integrity and reliability. The stress intensity factor, a vital parameter that describes the strength of the stress field at the crack tip, characterizes the level of stress concentration at the crack tip and directly correlates with the square root of the crack length. With a crack depth of 4 mm and crack lengths of 20, 37.5, 50, 62.5, and 75 mm, the effect of crack length on the driving force is minimal at a tensile strain of 0.2%, with the change in driving force being only about 0.008 mm. At a tensile strain of 0.5%, the driving force increases rapidly. The increase in the crack driving force for a crack length of 50 mm compared to 25 mm is 0.85 times that of the crack driving force for a crack length of 25 mm, while the increase for a crack length of 75 mm compared to 25 mm is 1.72 times (Figure 22). As the tensile strain rises, the relationship between the crack driving force and crack length remains nonlinear, with shorter crack lengths displaying greater sensitivity to changes. At a tensile strain of 3%, the growth in crack driving force from 25 to 75 mm increases by 0.751 mm, with 0.445 mm greater than the increase caused by a crack length ranging from 25 to 50 mm (Figure 23). As the tensile strain increases, the crack propagation driving force increases rapidly for shorter cracks, underscoring the importance of regular inspections of girth welds.

### 3.7. Crack Depth

**Figure 24:**
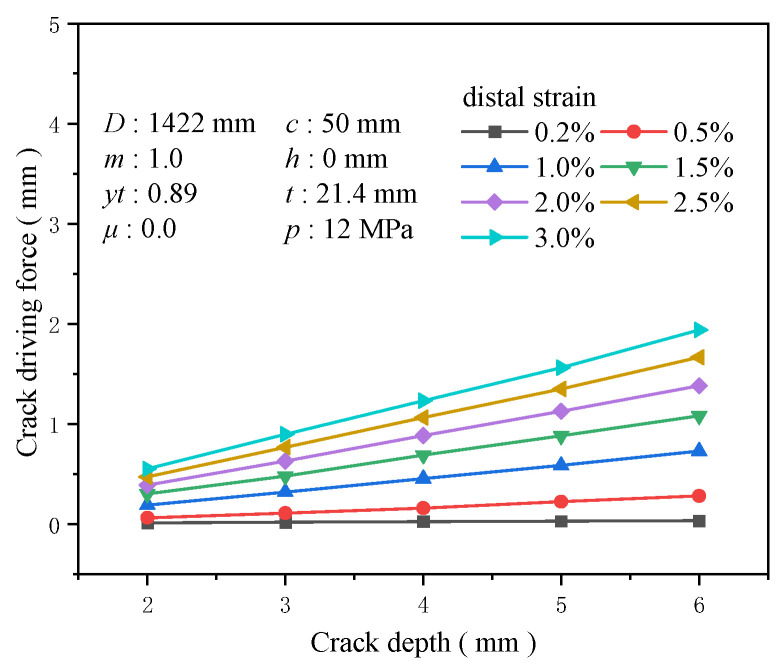
The crack driving force when the crack reaches various tensile strains under different crack depth conditions.

**Figure 25:**
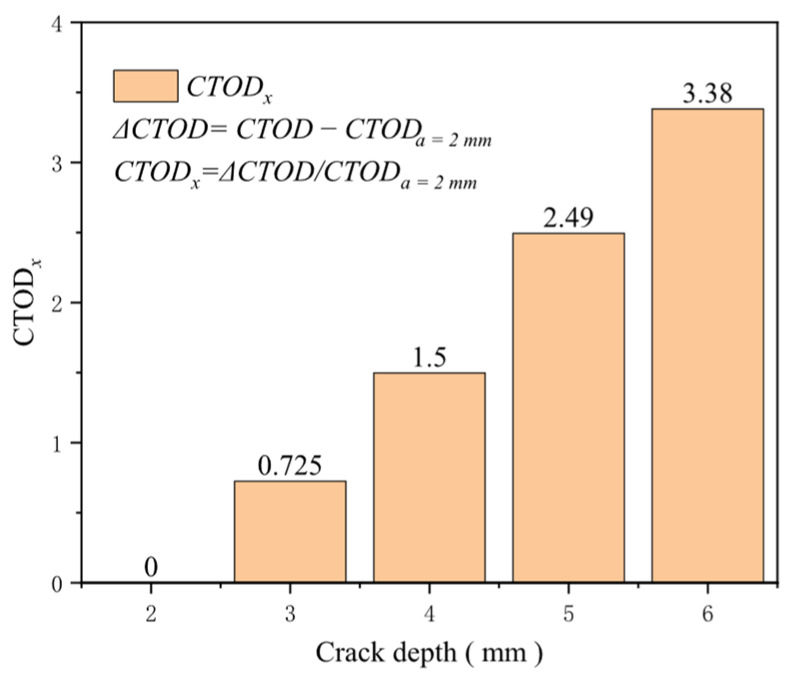
Under the condition of 0.5% distal strain, the change in crack depth causes an increase in driving force.

Similarly, crack depth is a crucial factor influencing the driving force of crack propagation. A larger crack depth provides a greater damaged area, exacerbating the stress concentration near the crack tip and increasing the likelihood of crack propagation. When maintaining the crack length at 50 mm and altering the crack depth to 2, 3, 4, 5, and 6 mm, crack depth emerges as one of the most sensitive factors affecting the crack driving force. At a tensile strain of 0.5%, the increase in the crack driving force caused by crack depths ranging from 2 to 6 mm is about 0.217 mm, and at a tensile strain of 3%, this growth is approximately 0.751 mm. Under identical tensile strain conditions, the sensitivity of the crack driving force to crack depth intensifies with increasing crack depth (Figure 24). At a tensile strain of 0.5%, the increase in the crack driving force for a crack depth of 4 mm compared to 2 mm reaches 1.5 times that of the crack driving force for a crack depth of 2 mm, while the increase for a crack depth of 6 mm compared to 2 mm is 3.38 times (Figure 25). Timely maintenance of crack defects is crucial for ensuring the safety of girth welds to the greatest extent possible.

### 3.8. HAZ Softening Rate

**Figure 26:**
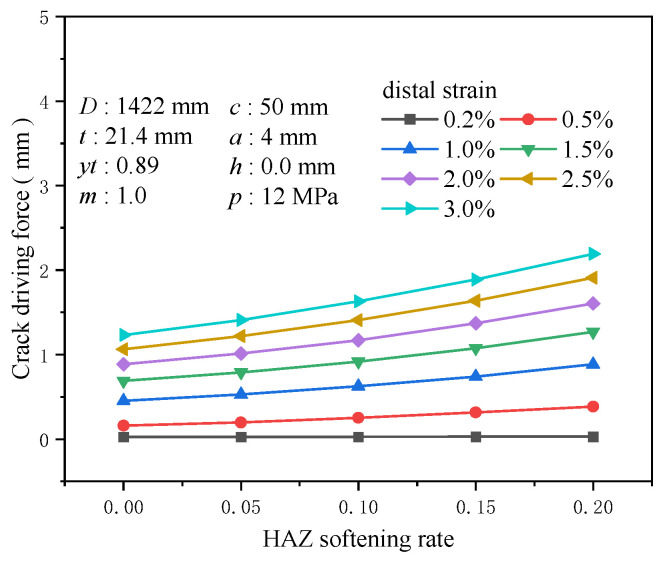
The crack driving force when the crack reaches various tensile strains under different HAZ softening rate conditions.

**Figure 27:**
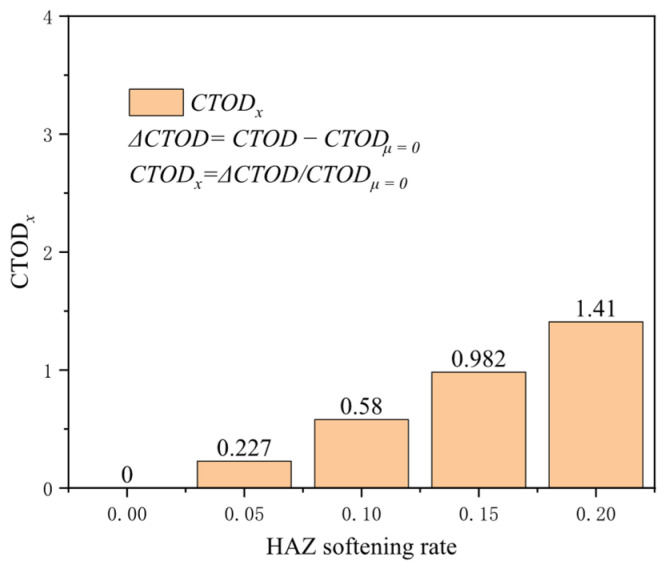
Under the 0.5% distal strain condition, the change in HAZ softening rate causes an increase in driving force.

The high temperatures during the welding process can impact the microstructure and mechanical properties of the base metal, resulting in variations in local hardness and strength, known as the HAZ. The reduced mechanical properties typically observed in the HAZ make it the primary area to bear loads under normal conditions. Setting the softening rate of the HAZ at 0, 0.05, 0.1, 0.15, and 0.2, the crack driving force exhibits high sensitivity to the softening rate, especially under extensive strain conditions. At a tensile strain of 0.2%, the increment in the crack driving force is minimal, about 0.004 mm; however, when the tensile strain rises to 0.5%, the growth in the crack driving force markedly increases to about 0.225 mm. At a 3% tensile strain, the increase is approximately 0.959 mm (Figure 26). The higher the degree of softening, the more significant the crack driving force induced by softening under the same strain conditions. At a tensile strain of 0.5%, the increase in the crack driving force for a softening rate of 0.2 compared to the non-softening condition is 1.41 times that of the crack driving force without softening (Figure 27). A high degree of softening significantly reduces material strength, rendering the HAZ more susceptible to plastic deformation under load. Therefore, accounting for the enhancing effect of the softening rate on crack propagation is crucial when managing crack propagation in girth welds.

### 3.9. Internal Pressure

**Figure 28:**
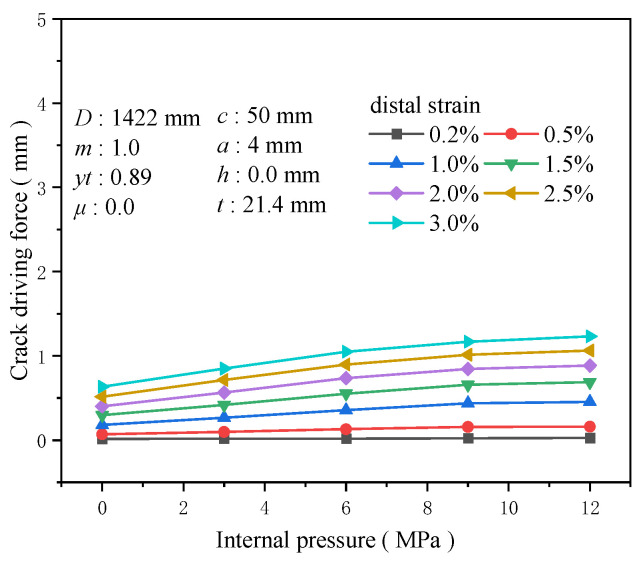
The crack driving force when the crack reaches various tensile strains under different internal pressure conditions.

**Figure 29:**
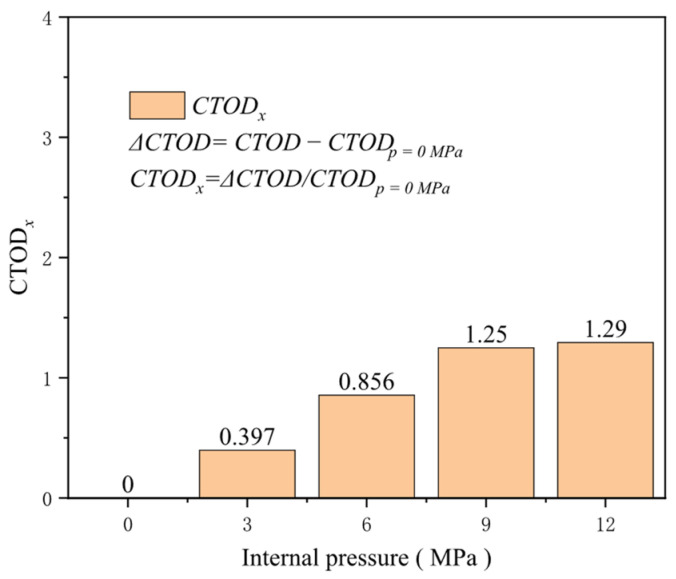
Under the condition of 0.5% distal strain, the change in internal pressure causes an increase in driving force.

Pipeline transportation typically operates under pressure, and the current trend is toward higher pressures. Compared to pipelines without internal pressure, internal pressure applies axial loads to the pipeline cross-section, effectively “pre-stretching” it to some extent and thus reducing its capacity to resist additional tensile loads. Keeping other factors constant and setting the internal pressure at 0, 3, 6, 9, and 12 MPa, the crack driving force in pipelines under varying internal pressure conditions shows a positive correlation with the internal pressure when the overall pipeline is subjected to a specific tensile strain. However, once the internal pressure exceeds 9 MPa, further increases in internal pressure have a less pronounced effect on enhancing the crack driving force. At a tensile strain of 0.5%, for internal pressures ranging from 0 to 9 in 3 MPa increments, the increase in the crack driving force is 0.027 mm for each interval, while the increase in the crack driving force for an internal pressure from 9 to 12 MPa is only 0.003 MPa (Figure 28). For low-pressure pipelines, the growth rate of the crack driving force is susceptible to the magnitude of internal pressure. At a tensile strain of 0.5%, the increase in the crack driving force for an internal pressure of 3 MPa compared to a pressure-less pipeline reaches 0.397 times that of the crack driving force without internal pressure. For an internal pressure of 6 MPa, the increase reaches 0.856 times that of the crack driving force without internal pressure (Figure 29). Therefore, in the actual safety assessment of cracked pipelines, the impact of internal pressure on crack driving forces cannot be overlooked. In addition, the inspection and maintenance of high-pressure pipelines should be more cautious and frequent to ensure their safe and stable operation throughout their service life.

### 3.10. Misalignment

**Figure 30:**
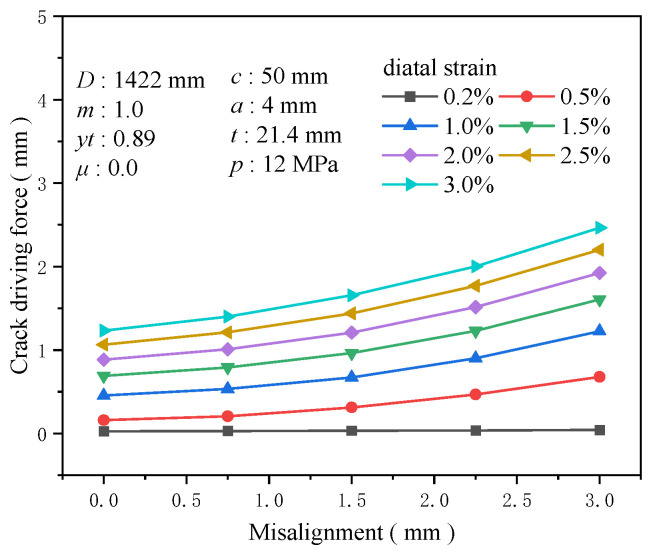
The crack driving force when the crack reaches various tensile strains under different misalignment conditions.

**Figure 31:**
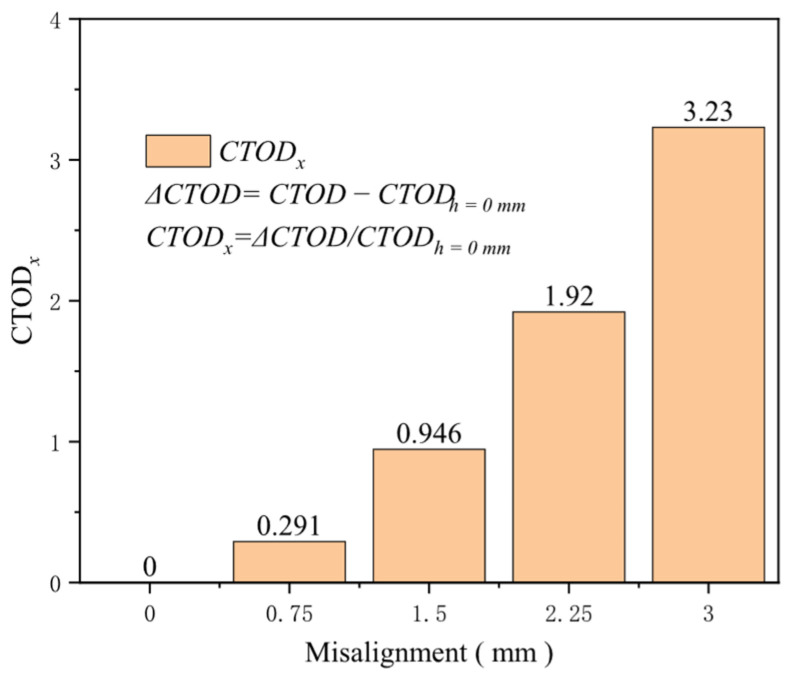
Under the condition of 0.5% distal strain, the change in misalignment causes an increase in driving force.

During the butt welding of pipelines, misalignment is inevitably present, which significantly reduces the load-bearing capacity of the girth weld. Therefore, many domestic and international standards have set critical values for the amount of misalignment to mitigate risks. This research set the misalignment sizes to 0, 0.75, 1.5, 2.25, and 3 mm. When analyzing different tensile strains, it is observed that at a 0.2% tensile strain, the impact of misalignment on the crack driving force is minimal. However, as the tensile strain increases, the crack driving force becomes highly sensitive to the misalignment (Figure 30). Comparisons between girth welds with and without misalignment reveal that the crack driving force is profoundly affected by variations in misalignment; after the misalignment exceeds 2 mm, there is a marked escalation in the crack driving force. At a 0.5% tensile strain, the increase in the crack driving force from a misalignment of 0 to 3 mm is 0.517 mm, with 0.262 mm attributable to misalignments exceeding 2 mm. In addition, at a misalignment of 1.5 mm, the augmentation in the crack driving force relative to no misalignment is 0.946 times the crack driving force without misalignment. At a misalignment of 3 mm, the increase reaches 3.23 times the initial force (Figure 31). Misalignment diminishes the effective geometric cross-sectional area at the girth weld location, intensifies stress concentration there, and reduces the ultimate strain capacity of the girth weld. Accordingly, misalignment diminishes the overall performance of the welded joint and exerts an increasingly adverse impact as the degree of misalignment rises. Hence, strictly controlling the degree of misalignment during design and construction is imperative for ensuring the structural integrity and reliability of the girth weld.

## 4. Establishment and Accuracy Verification of Prediction Model

### 4.1. Prediction Model Based on Light Gradient Boosting Machine

#### 4.1.1. Light Gradient Boosting Machine

**Figure 32:**
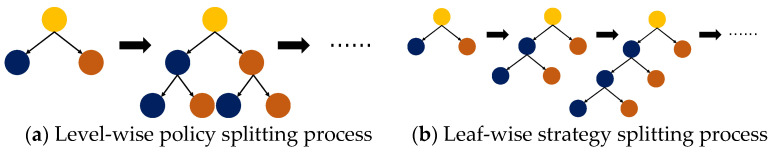
LightGBM split diagram.

The LightGBM algorithm represents an efficient improvement over the XGBOOST algorithm proposed by Microsoft [28]. In traditional Gradient Boosting Decision Tree (GBDT) algorithms, the level-wise strategy splits all nodes at the current level, leading to many ineffective splits and reduced computational efficiency. LightGBM employs a leaf-wise strategy, selecting the node with the highest gain for splitting, which reduces unnecessary splits and enhances computational efficiency (Figure 32) [29,30]. LightGBM introduces unilateral gradient sampling and mutually exclusive feature bundling to achieve fast calculation and improve the model’s fitting effect. Unilateral gradient sampling is a sampling algorithm employed to retain data with larger gradients and remove data with smaller gradients, improving the training effect of the model and accelerating the training speed. The mutual exclusion feature bundling algorithm can reduce the dimension of features without loss, bind different features together, merge multiple features into a single feature, and effectively reduce the number of features [31].

The objective function of LightGBM is mainly composed of the loss function and regularization term. The objective function of LightGBM can be expressed as:

(7) $$Objection=\sum_{i}^{n}l\left(y_{i},{{\hat{y}}_{i}}^{t-1}+f_{t}(x_{i})\right)+\Omega (f_{t})$$

where $y_{i}$ is the true value of the *i* sample, ${{\hat{y}}_{i}}^{t-1}$ is the predicted value of the first t − 1 trees for the *i* sample, $l\left(y_{i},{\hat{y}}_{i}\right)$ is the loss function between the true and predicted values, and $f_{t}$ is the model of the *t* tree.

(8) $$\Omega \left(f_{t}\left(x\right)\right)=\gamma T+1/2\lambda \sum_{t=1}^{T}{w_{t}}^{2}$$

where $\sum \Omega (f_{t})$ is the complexity of the first t trees, γ is the leaf node coefficient, T is the number of leaf nodes, and γ is the L2 regularization coefficient.

The objective function can be obtained by using the second-order Taylor expansion, merging and simplifying as follows:

(9) $$Objection=\sum_{i=1}^{n}\left[l\left(y_{i},{{\hat{y}}_{i}}^{t-1}\right)+g_{i}f_{t}\left(x_{i}\right)+1/2h_{i}f_{t}^{2}\left(x_{i}\right)\right]+\Omega \left(f_{t}\right)$$

where $g_{i}=\partial l\left(y_{i},{{\hat{y}}_{i}}^{t-1}\right)/\partial {{\hat{y}}_{i}}^{t-1}$ and $h_{i}={\partial^{2}l\left(y_{i},{{\hat{y}}_{i}}^{t-1}\right)/\partial {{\hat{y}}_{i}}^{t-1}}^{2}$.

Further simplification of (9) leads to (10).

(10) $$Objection=\sum_{t=1}^{T}\left[G_{t}w_{t}+1/2\left(H_{t}+\lambda \right){w_{t}}^{2}\right]+\gamma T$$

where $G_{t}=\sum_{i\in I_{t}}g_{i}$ and $H_{t}=\sum_{i\in I_{t}}h_{i}$.

The partial derivative of $w_{t}$ is made to be 0, and (10) is further obtained.

(11) $$w_{t}=-G_{t}/H_{t}+\lambda$$

Bring (11) into (10) to get the final objective function (12):

(12) $$Objection=-1/2\sum_{t=1}^{T}G_{t}^{2}/(H_{t}+\lambda )+\gamma T$$

#### 4.1.2. Prediction Model Establishment

Based on the calculation results for the crack driving force from 10,935 sets of full-size pipeline girth welds, calculated using a data matrix, the LightGBM machine learning algorithm is utilized to construct a model for predicting crack driving forces. The database size determines the range of influence factors for this model, as specified in Table 1. To effectively evaluate the developed model, the dataset was divided into training and testing sets using a 9:1 ratio. To ensure the validity of model evaluation, the training and testing datasets were strictly separated. Furthermore, to guarantee the consistency and comparability of results, the same training and testing sets were used throughout the entire study for all model analyses and comparisons.

Due to the black-box nature of machine learning algorithms, a penalized least squares loss function is adopted to ensure the safety of pipeline girth welds. This function is described in Equation (13).

(13) $$Obj=\left\{\begin{array}{l} {\left(y_{i}-{\hat{y}}_{i}\right)}^{2}/1+y_{i},y_{i}-{\hat{y}}_{i}\geq 0\\ 10\times {\left(y_{i}-{\hat{y}}_{i}\right)}^{2}/1+y_{i},y_{i}-{\hat{y}}_{i}<0 \end{array}\right.$$

where the value of the i th sample in the test set is $y_{i}$, and the CTOD prediction value of the i th sample in the test set is ${\hat{y}}_{i}$.

**Figure 33:**
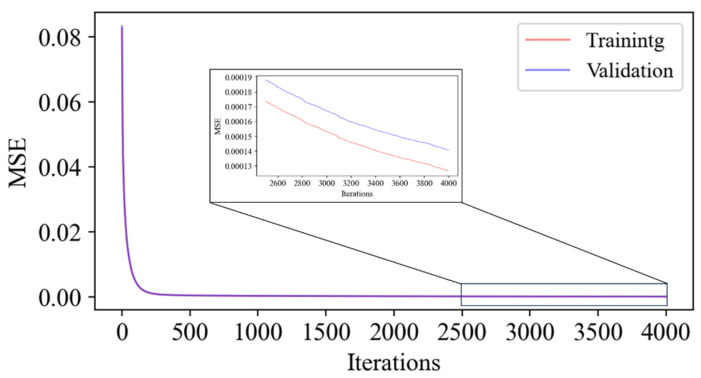
Model training process.

Based on Equation (13), the model’s prediction is deemed less conservative when the predicted CTOD value exceeds the actual CTOD value. A penalty coefficient is introduced to the loss function to penalize the model, thus increasing the loss function’s value. When the predicted CTOD value is less than the actual CTOD value, the model’s prediction is considered more conservative, and no penalty is applied, leaving the loss function unchanged. The model’s predictions are biased toward lower values than the actual values, ensuring the conservatism of the predictions by implementing a penalized least squares loss function. Simultaneously, this study adopts the L2 regularization method and sets the L2 regularization coefficient to 0.05 to mitigate the risk of overfitting (Figure 33).

The R^2^ of the model’s test set predictions was 0.99692, the MSE was 0.00122, and the MAPE was 8.943%. Since the R^2^ is nearly 1 and the MAPE between the test set predictions and the actual values is below 10%, the model exhibits an excellent fit and accurately reflects the relationship between feature values and the crack driving force [32]. To further quantify the model’s error behavior in CTOD units, the Root Mean Square Error (RMSE) is additionally calculated based on the test set data. The result shows that the RMSE of the model in CTOD units is 0.078 mm, indicating that the absolute deviation between the predicted and true CTOD values is controllable.

**Figure 34:**
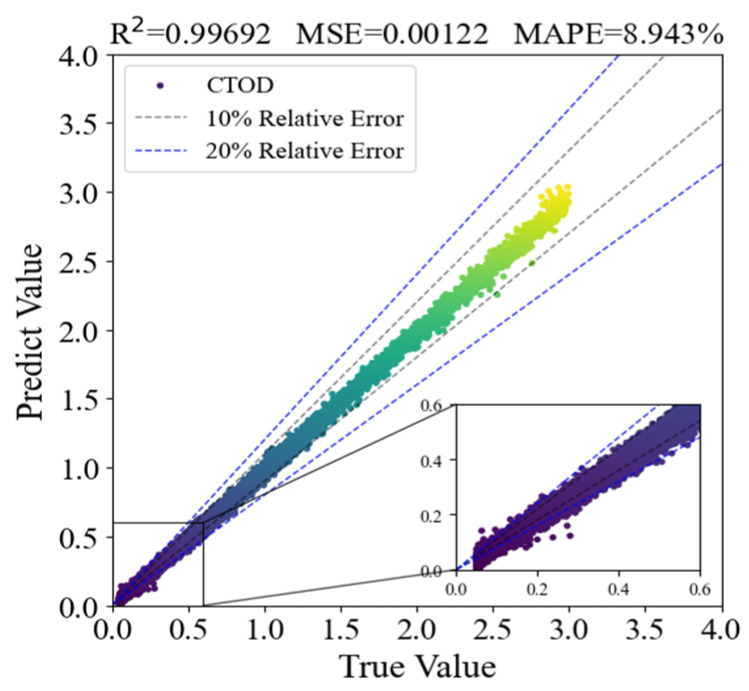
Error diagram of OBJ-LightGBM model test results.

Notably, the model’s error behavior exhibits certain differences across CTOD value ranges: when predicting smaller CTOD values, the relative error between the predicted and actual values can exceed 20% in some cases, indicating relatively lower accuracy for this range; in contrast, when predicting larger CTOD values, the relative error is usually below 10%, demonstrating good accuracy (Figure 34). However, from the perspective of absolute error, the predicted absolute error of small CTOD values is all less than 0.08 mm, which is much lower than the critical judgment threshold of crack driving force in engineering practice, thus not affecting the practical application of the model.

**Figure 35:**
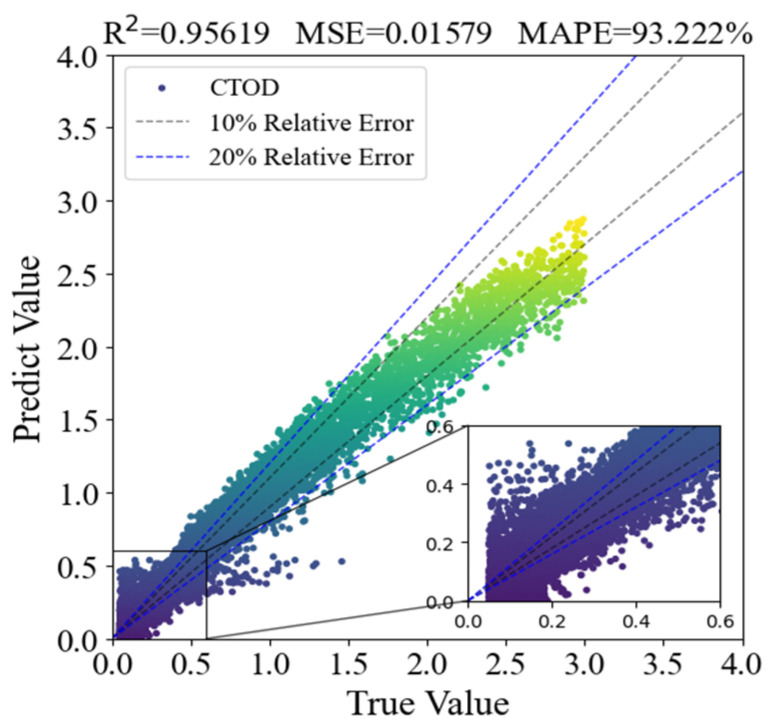
Error diagram of LightGBM model test results.

The test results of LightGBM without the penalized least squares loss function are shown in Figure 35, with an R^2^ of 0.95619, an MSE of 0.01579, and an MPAE of 93.222%. Therefore, the penalized least squares method significantly enhances the model’s fitting performance.

#### 4.1.3. Model Interpretability Analysis Based on SHAP

SHAP (SHapley Additive exPlanations) analysis provides an in-depth interpretation of the role of each input feature by precisely computing the SHAP values for individual samples. This method quantifies the independent contribution of each feature to the prediction outcome, thereby offering a comprehensive perspective for understanding the model’s decision-making process [26]. The formula for calculating SHAP values is presented in Equation (11). After computing SHAP values for all samples, the absolute values are averaged across the dataset to obtain the importance ranking of different features.

(14) $${\phi_{i}}^{\left(k\right)}=\sum_{S\subseteq F}\frac{\left|S\right|!\left(\left|F\right|-\left|S\right|-1\right)!}{\left|F\right|!}\left[f\left(S\cup \left\{i\right\}\right)-f\left(S\right)\right]$$

In the SHAP formulation, *Φ_i_*^(*j*)^ represents the SHAP value of the *i*-th feature for the *j*-th individual sample. *S* denotes any subset of the feature set *F* that does not contain the *i*-th feature; ∣*S*∣ is the number of elements in subset *S*; ∣*F*∣ is the total number of features. *f*(*S*) represents the model’s prediction when only the features in subset *S* are included, and *f*(*S*∪{*i*}) is the prediction when the *i*-th feature is added to subset *S*.

**Figure 36:**
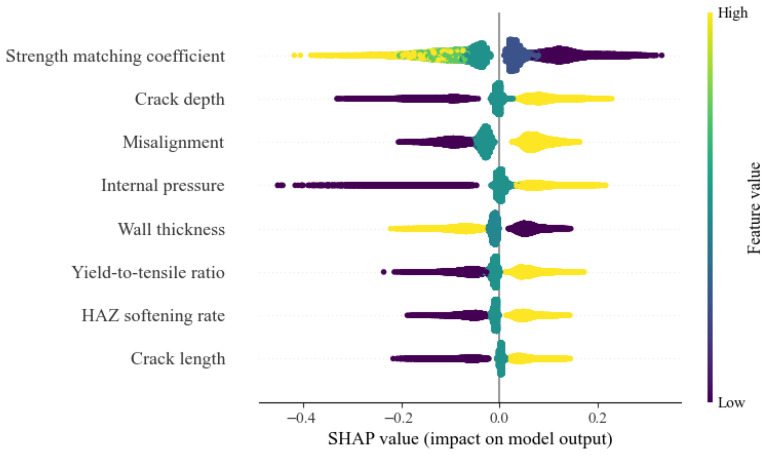
SHAP model interpretation for CTOD prediction model.

The SHAP-based interpretability results of the developed model are presented. The results indicate that the strength matching coefficient, crack depth, and misalignment are the dominant factors governing the CTOD of cracked pipelines. Specifically, an increase in the strength matching coefficient exerts a pronounced negative effect on the predicted CTOD, leading to a reduction in CTOD values (Figure 36). A similar adverse influence is observed for wall thickness, where larger wall thickness levels correspond to lower CTOD predictions. In contrast, crack depth and misalignment exhibit a clear positive contribution to the predicted CTOD, with larger crack depths and misalignment resulting in increased CTOD values. In addition, internal pressure, yield-to-tensile strength ratio, HAZ softening rate, and crack length show trends consistent with that of crack depth, all contributing positively to CTOD, although their effects are comparatively less significant than those of the dominant factors.

### 4.2. Prediction Model Accuracy Validation

**Figure 37:**
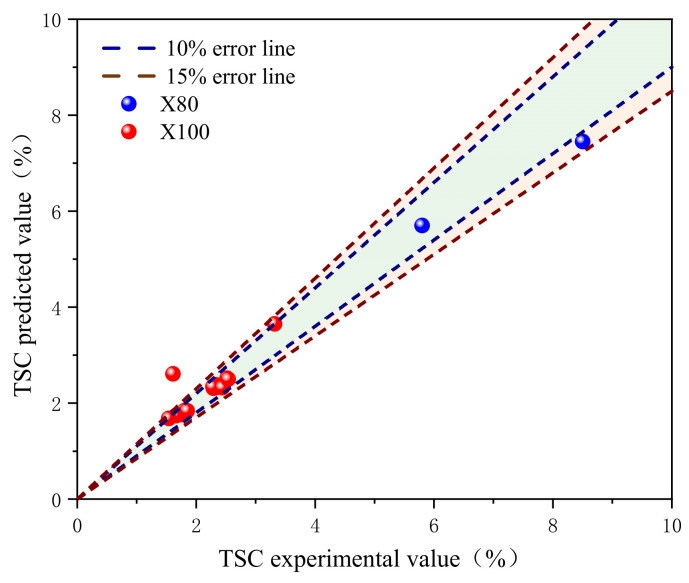
Verification results of girth weld strain capacity prediction model.

This study analyzed 18 sets of wide-plate test data of X80 and X100 pipelines to validate the crack driving force prediction model [33,34] (Appendix A). The model’s predictions are closely aligned with the actual TSC experimental data. The average error of the 18 tests is 6.5%, and except for one X100 test which had a relatively larger error, the errors of all other tests are within 15% (Figure 37). These results clearly demonstrate the validity and accuracy of the model in predicting the strain capacity of high-grade steel pipelines.

## 5. Conclusions

Based on the secondary development of finite element simulation, this study conducts a sensitivity analysis of the factors influencing the crack driving force in pipeline girth welds and identifies the most significant factors. A prediction model is developed using machine learning algorithms that offer broader application parameters and enhanced efficiency. The primary conclusions are as follows:

1. Key data such as load–displacement curves and strain distribution obtained from the full-scale four-point bending–internal pressure combined load test were compared with the calculation results of the established finite element model of D1219 × 22 mm X80 pipeline girth weld with cracks for verification. The results indicate that the modeling method adopted in this study can accurately capture the mechanical behavior of the pipeline and crack evolution characteristics, and the established finite element model has good accuracy.
2. The size of defects and material properties influence the crack driving force more than structural factors. The most influential factors affecting the crack driving force are the crack depth, strength matching coefficient, and misalignment. At a tensile strain of 0.5%, with other factors held constant, the increase in the crack driving force due to a low strength matching coefficient reached 2.6 times that of equivalent strength matching. Similarly, an increase in the crack driving force caused by a 3 mm misalignment reached 3.23 times that of no misalignment, and an increase in the crack driving force when the crack depth was 6 mm, compared to 2 mm, reached 3.38 times that of a 2 mm crack depth. The key to the precision of the semi-empirical formula lies in identifying the factors that enhance the growth of the crack driving force. In addition, controlling these factors can significantly increase the strain-bearing capacity of the girth weld. Prioritizing high-strength matching in girth welds is crucial. Simultaneously, advancements in welding technology are essential to minimize misalignment, and enhancements in monitoring capabilities are vital for detecting and controlling cracks at their inception.
3. When utilizing influencing parameters such as the strength matching coefficient, softening rate, yield strength ratio, crack length, crack depth, wall thickness, misalignment, and internal pressure, a total of 10,935 sets of full-scale pipeline girth weld crack driving force calculation results are generated using the parameter matrix method. A crack driving force database is established, and a prediction model is developed using the LightGBM machine learning model. Since the finite element calculation results of the crack driving force are not sensitive to tensile strength, the prediction model is applicable across various steel grades.
4. The developed prediction model is employed to predict the outcomes of 18 sets of wide-plate tests. The results indicated that the proposed strain capacity prediction model demonstrated significant advantages for girth welds with higher strain capacity.
5. The established prediction model has a clear validity envelope as defined in Table 1, and its application is limited to the specified parameter ranges. Further verification is required to assess its applicability to scenarios beyond these parameter ranges.

## Figures and Tables

**Table 1:** Prediction model value condition setting table.

Factors	Value Ranges
Wall thickness (*t*)	21.4–30.8 mm
Crack depth (*a*)	2–6 mm
Crack length (*c*)	25–75 mm
Misalignment (*h*)	0–3 mm
Internal pressure (*p*)	0–12 MPa
Strength matching coefficient (*m*)	0.8–1.2
HAZ softening rate (*μ*)	0–0.2
Yield-to-tensile ratio (*yt*)	0.85–0.93
Crack location	The centerline of the inner surface of the pipeline

## Data Availability

The original contributions presented in the study are included in the article, further inquiries can be directed to the corresponding author.

## Appendix A. 18 Sets of Wide-Plate Test Data

**No.**	**Steel Grade**	**Defect Location**	**Geometric Parameters**					**Strength Parameters**		**Load Parameters**		**CTODA**	**TSC Test Value**	**TSC Predict Value**
			**D**	**t**	**a**	**2c**	**H** **(** **Misalignment** **)**	**Y/T**	**OM**	**fp**	**uEL**	**From CVN**		
			**(mm)**	**(mm)**	**(mm)**	**(mm)**	**(mm)**	**(ksi/ksi)**	**(ksi/ksi)**	**(ksi/ksi)**	**(%)**	**(mm)**	**(%)**	**(%)**
1	X80	WM	1219.00	25.40	3.00	63.70	0.00	0.94	1.32	0.00	6.50	0.67	5.80	5.70
2	X80	WM	1219.00	25.40	6.00	31.80	0.00	0.92	1.38	0.00	6.50	0.89	8.50	7.45
3	X100	WM	1219.00	19.80	5.00	63.70	0.00	0.89	1.14	0.00	6.00	0.48	1.80	1.84
4	X100	WM	1219.00	19.80	4.00	31.80	0.00	0.89	1.16	0.00	6.00	0.48	2.30	2.32
5	X100	WELD	914.00	19.10	3.00	50.00	0.00	0.91	1.06	0.00	6.00	0.69	2.33	2.38
6	X100	WELD	914.00	19.10	3.14	50.00	0.00	0.91	1.06	0.00	6.00	0.69	2.52	2.52
7	X100	WELD	914.00	19.10	3.27	50.00	0.00	0.91	1.07	0.00	6.00	0.69	2.29	2.31
8	X100	WELD	914.00	19.10	6.00	30.00	0.00	0.91	1.08	0.00	6.00	0.69	1.83	1.81
9	X100	WELD	914.00	19.10	6.06	30.00	0.00	0.91	1.06	0.00	6.00	0.69	1.76	1.76
10	X100	WELD	914.00	19.10	2.12	75.00	0.00	0.91	1.07	0.00	6.00	0.69	2.33	2.32
11	X100	WELD	914.00	19.10	2.23	75.00	0.00	0.91	1.07	0.00	6.00	0.69	3.32	3.65
12	X100	WELD	914.00	19.10	2.95	50.00	0.00	0.91	1.06	0.00	6.00	0.69	2.28	2.34
13	X100	WELD	914.00	19.10	3.21	50.00	0.00	0.91	1.06	0.00	6.00	0.69	2.44	2.32
14	X100	WELD	914.00	19.10	2.88	50.00	0.00	0.91	1.03	0.00	6.00	0.61	1.66	1.74
15	X100	WELD	914.00	19.10	2.96	50.00	0.00	0.93	1.03	0.00	6.00	0.61	1.85	1.84
16	100	WELD	914.00	19.10	6.36	30.00	0.00	0.93	1.04	0.00	6.00	0.61	1.54	1.68
17	100	WELD	914.00	19.10	6.15	30.00	0.00	0.93	1.04	0.00	6.00	0.61	1.61	2.61
18	100	WELD	914.00	19.10	3.35	50.00	0.00	0.93	1.03	0.00	6.00	0.61	2.54	2.50
